# Rating scales to inform balance exercise difficulty during rehabilitation for individuals with neurological disorders

**DOI:** 10.3389/fresc.2026.1703537

**Published:** 2026-03-18

**Authors:** Brooke N. Klatt, Tian Bao, Kathleen H. Sienko, Carrie W. Hoppes, Patrick J. Sparto, Susan L. Whitney

**Affiliations:** 1 Department of Physical Therapy, University of Pittsburgh, Pittsburgh, PA, United States; 2 Department of Mechanical Engineering, University of Michigan, Ann Arbor, MI, United States; 3 Department of Biomedical Engineering, University of Michigan, Ann Arbor, MI, United States; 4 Advanced Exposures, Diagnostics, Interventions, and Biosecurity (AEGIS) Program, Joint Base San Antonio-Lackland Airforce Base, San Antonio, TX, United States

**Keywords:** balance, exercise prescription, physical therapy, rating scale, rehabilitation

## Abstract

Appropriate exercise dosing is essential for optimizing functional outcomes in rehabilitation for individuals with neurological disorders. While guidelines for aerobic and resistance training intensity are well established, comparable standards for balance exercise remain limited, despite its central role in reducing falls and promoting safe mobility. Clinically feasible approaches to quantifying balance exercise difficulty are needed to support safe progression and individualized prescription. This exploratory study explored the relationship between participants perceived balance difficulty and clinician-rated assistance during progressive balance training. Sixteen adults (68.1 ± 8.0 years old; 69% female) with balance disorders (peripheral vestibular hypofunction, peripheral neuropathy, or age-related balance impairment) completed an 18-session balance rehabilitation program over 6-weeks. Participants and clinicians rated each exercise using standardized scales, yielding 1,658 rating comparisons. Agreement between participant-perceived difficulty and clinician-rated assistance was moderate [Cohen's kappa (*k*) = 0.42, 59% exact agreement*, p* < 0.001], with higher agreement observed using weighted analysis (linear weighted *k* = 0.58; quadratic weighted *k* = 0.74, both *p* < 0.001). Most rating discrepancies (85%) differed by only one category, suggesting clinically minor disagreements. These preliminary findings indicate that participant-perceived difficulty may provide a clinically accessible indicator of balance exercise challenge. However, validation in larger samples with independent rating procedures and examination of relationships between difficulty ratings and functional outcomes are needed before clinical implementation. This work provides initial evidence for incorporating participant feedback into balance exercise prescription as part of individualized, patient-centered rehabilitation approaches.

## Introduction

Prescribing an exercise program that is of an appropriate intensity to achieve functional improvements is important for all people engaging in an exercise program, particularly for children and adults living with neurological disorders who may experience reduced mobility, strength, and participation in daily life. The American College of Sports and Medicine has clearly defined how to measure intensity using heart rate reserve for aerobic activity and one repetition maximum measurement is used to measure intensity for resistance exercise (1). Additionally, scales that rate perceived exertion have successfully been used to record intensity based on the subjective report from the person completing the activity. Intensity measures can then be used to guide the prescription of exercises aiming to achieve optimal outcomes.

Unlike aerobic and resistance intensity exercise guidelines, balance exercise guidelines are not well defined (1, 2). This gap is significant given that balance training is a cornerstone of therapeutic exercise for neurological rehabilitation, with the potential not only to reduce fall risk but also to enhance functional independence, participation, and quality of life. Some investigators have attempted to use center of pressure displacement to measure balance intensity while progressively challenging subjects with more difficult standing balance conditions that alter the visual and somatosensory inputs (3). While this might be helpful in ordering exercises based on level of difficulty, clinical usability is limited as many physical therapists do not have the resources to record center of pressure within their clinical practice.

Others have aimed to measure balance intensity by observing non-verbal responses of individuals such as postural reactions (ankle, hip, and stepping strategies), bracing (stiffening of muscles groups), postural sway, and breathing changes (increased depth and/or rate) during balance exercises (4). Measuring balance intensity through non-verbal responses can be challenging for many reasons including clinician subjectivity and the lack of standardized, validated methods for observation and documentation. Developing clinically feasible, individualized methods for quantifying balance exercise intensity is therefore essential to advance precision exercise dosing, promote safety, and support the long-term goal of restoring activity and participation for individuals with neurological disorders.

In this study, we operationalize balance exercise intensity through two indirect but clinically accessible measures: participant-perceived difficulty and clinician-rated assistance. While these constructs differ from direct physiological measures of exercise intensity (e.g., metabolic demand), they represent practical indicators of task difficulty that can inform clinical decision-making. Our group previously investigated two rating scales of perceived difficulty for balance exercises and found them to have moderate to strong correlations with kinematic postural stability measures (5). In that investigation, the participants verbally provided numeric ratings that reflected their perceived difficulty during different balance exercises. This verbal report is clinically feasible as a clinician can quickly and easily record a numeric rating within a treatment session. Another factor that might be useful to determine the difficulty of a balance exercise is the amount of assistance the participant requires from the supervising clinician. Level of assistance is frequently documented in rehabilitation clinical practice using terms such as independent, supervision, minimal assistance, moderate assistance, and maximal assistance as modeled from the Functional Independence Measure (FIM) (6).

This study aimed to determine whether participant-perceived balance difficulty corresponds with clinician-rated assistance level during progressive balance training in individuals with neurological and balance disorders. Establishing this relationship is important because participant feedback has the potential to inform individualized balance exercise prescription and safe progression. We hypothesized that participant ratings of perceived balance difficulty would correlate with clinicians' ratings of assistance provided.

## Methods

This was a prospective observational analysis conducted as a secondary aim within a randomized controlled trial (ClinicalTrials.gov: NCT02867683). The parent trial examined the effects of vibrotactile feedback on balance outcomes in individuals with vestibular and balance disorders. The current analysis focuses on agreement between participant-perceived balance difficulty and clinician-rated assistance during progressive balance training, independent of treatment group assignment. Although the sample included heterogeneous diagnoses with different underlying mechanisms of balance impairment (unilateral vestibular hypofunction, bilateral vestibular hypofunction, peripheral neuropathy, and older adults with self-reported balance impairment), participants were analyzed as a single cohort to evaluate the rating system's feasibility across diverse clinical presentations representative of typical balance rehabilitation practice. Subgroup analyses by diagnosis were not conducted due to limited sample sizes within diagnostic categories.

### Participants

Adults aged 21–80 years old with balance disorders including diagnoses of unilateral peripheral vestibular hypofunction, bilateral peripheral vestibular hypofunction, or peripheral neuropathy were recruited to participate in the study. Additionally, older adults, age 60 and above, who reported balance impairment were recruited for participation. Balance impairment was defined as self-reported episodes of balance instability or loss of balance occurring during daily activities. Study recruitment flyers were placed in vestibular neurology and physical therapy clinics and the study was also advertised on the University of Pittsburgh Research Participant Registry. This study was approved by the Institutional Review Board (IRB) at the University of Pittsburgh. All procedures were conducted in accordance with the ethical guidelines set forth by the IRB, and participants provided informed consent prior to their participation in the study.

A Montreal Cognitive Assessment (MOCA) (7) score of ≥26, bilateral ankle dorsiflexion active range of motion ≥10 degrees, and bilateral ankle plantarflexion active range of motion ≥20 degrees were required for eligibility. Ankle range of motion criteria ensured adequate mobility for weight-shifting and dynamic balance tasks requiring ankle postural control strategies. Exclusionary criteria included confounding neurologic or neuromuscular disorders, pregnancy, inability to stand for 3 min without rest, recent lower extremity fracture/severe pain within the last six months, previous lower extremity joint replacement, incapacitating back or lower extremity pain, and a person whose waist circumference >127 cm.

### Balance training

Each of the participants completed a balance training program consisting of 18 training sessions lasting 45–60 min over the course of 6 weeks. The program followed a published conceptual framework that includes a ranked ordering of exercises in six different categories (standing on firm surface, standing on foam surface, weight shifting, modified center of gravity, gaze stabilization, and gait) (8). Within each of the six categories, exercises are ordered from least to most difficult as different variables are manipulated to alter the difficulty of the exercise within each exercise category, allowing individualization and progression across sessions based on participant performance and tolerance. The published balance training protocol represents a structured progression that was created in collaboration with experienced clinicians. While the systematic progression framework was created for research purposes, exercise types and progression principles were designed to reflect clinical practice in vestibular and balance rehabilitation.

**Figure 1:**
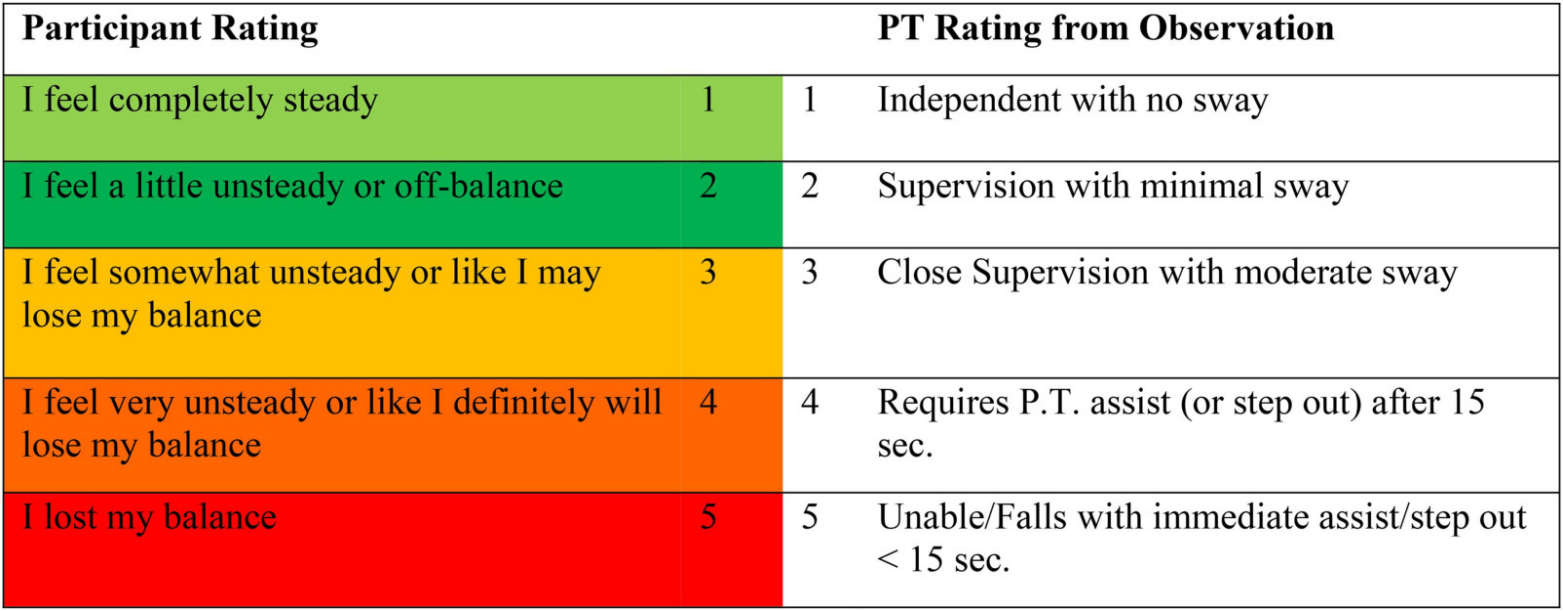
Balance rating scale for the participant and the physical therapist.

Within every training session, participants completed five or six balance exercises each session (five exercises/session for those with peripheral neuropathy and six exercises/session for the other three diagnostic groups). Participants with peripheral neuropathy did not complete the gaze stabilization exercises. Each exercise consisted of six trials lasting 30 s. Following every exercise, the physical therapist first recorded the assistance rating. The participant then independently recorded their balance rating for that exercise. The participant rating scale was adapted from a scale developed at the Cleveland Clinic (9) and the physical therapist rating scale was adapted from the FIM (6) (Figure 1). The physical therapist recorded their assistance rating immediately upon completing each exercise, prior to obtaining the participant's perceived difficulty rating, ensuring independence of ratings for each exercise trial. The participant was not aware of the numerical rating assigned by the physical therapist. Following each session, the study team reviewed both sets of ratings to inform exercise selection and progression decisions for the subsequent session. Thus, ratings served dual purposes: to assess agreement between participant and clinician perceptions, and to guide clinical progression decisions. All balance training sessions and ratings were provided by a single physical therapist with over 10 years of clinical experience designation as an ABPTS board-certified specialist in neurologic physical therapy.

During balance training, participants could rest as needed. To objectively assess fatigue, each session began and ended with two trials of a normalization exercise (semi-tandem stance, eyes closed, 30 s) to compare perceived balance performance by the participant and assistance rating by the physical therapist (PT). Exercises were individualized, with progression based on prior performance using research-defined rules. Initial starting levels were determined by a board-certified neurologic clinical specialist with 12 years of experience. Progression decisions for subsequent sessions were based on the participant and PT rating scale (Figure 1), prioritizing the PT's assessment if ratings differed. If the participant and the PT rated an exercise at a “1” or a “2”, the participant was progressed two levels within that exercise category the next session. If the participant and the PT rated the exercise as a “3”, the exercise was repeated for that category during the next session. Participants were moved back one level to an easier exercise within the category if the participant and PT rated the exercise at a “4” or a “5”. If the participant and PT ratings differed, the PT rating was used to determine the progression.

### Statistical analysis

To investigate the inter-rater agreement between the PT and the participant's balance ratings a Cohen's kappa coefficient was calculated for the rating comparisons (10). The Cohen's kappa is a more vigorous measure of agreement than a basic percent agreement calculation as it accounts for the occurrence of agreement from chance, which was also calculated. We interpreted kappa values using established guidelines (11): 0.41–0.60 (moderate), 0.61–0.80 (substantial), and >0.80 (near-perfect agreement). Moderate or higher agreement (*κ* ≥ 0.41) was considered potentially clinically meaningful for guiding exercise progression.

Linear and quadratic weighted kappa analysis was performed to account for disagreements between the two raters, and Stata® Statistical Software was used to analyze the relationship between the participant and the PT ratings. The penalty for being off by each category, or rating, is the same in the linear weighting. In the quadratic weighting, the penalty becomes greater as the separation between ratings increases (12). A quadratic-weighted coefficient compares the variability between pairs of items to the total variability and is equal to the intra class correlation coefficient in a two-way analysis of variance (13, 14).

## Results

### Sample characteristics

The sample included 16 participants including seven people with unilateral vestibular hypofunction, two people with bilateral vestibular hypofunction, four people with peripheral neuropathy, and three older adults (age 60 and above) with self-reported balance dysfunction (Table 1).

**Table 1:** Study participant demographics.

Participant characteristics	Unilateral vestibular hypofunction (*n* = 7)	Bilateral vestibular hypofunction (*n* = 2)	Peripheral neuropathy (*n* = 4)	Older adult with balance disorder (*n* = 3)	Total (*n* = 16)
Female % (*n*)	78% (*n* = 7)	100% (*n* = 3)	50% (*n* = 2)	33% (*n* = 1)	69% (*n* = 11)
Age (mean ± SD)	66 years ± 10.0	76.1 years ± 2.6	68.4 years ± 2.0	68.2 years ± 9.2	68.1 years ± 8.0

### Total rating comparisons

A total of 1,658 rating comparisons were collected from the 16 participants in our sample. Participants with unilateral vestibular hypofunction, bilateral vestibular hypofunction, or age-related balance impairment completed all six exercise categories across 18 sessions, yielding an expected 108 total rating comparisons. Three participants obtained 106 or 107 comparisons due to administrator error, and two participants obtained 110–111 comparisons because exercises resulting in immediate falls were replaced with less difficulty exercise alternatives. Participants with peripheral neuropathy completed five exercise categories (excluding gaze stabilization exercises), yielding an expected 90 rating comparisons per participant. One participant with peripheral neuropathy obtained 91 rating comparisons due to exercise substitution following immediate falls.

### Overall agreement statistics

A Cohen's kappa (*k*) = 0.42 (*P* < 0.001) with a 59% agreement between the PT and the participant balance ratings was recorded (Table 2). Percent agreement represented the proportion of rating comparisons with identical numerical ratings between participant and clinician.

**Table 2:** Percent agreement and Cohen's kappa for each participant and for entire sample **.**

Subject ID	Percent agreement	Cohen's Kappa (*k*)	*P*
UVH 1	55.1%	.394	<.001
UVH 2	61.3%	.478	<.001
UVH 3	60.4%	.397	<.001
UVH 4	45.4%	.122	.043
UVH 5	56.5%	.321	<.001
UVH 6	65.7%	.410	<.001
UVH 7	68.5%	.542	<.001
BVH 1	49.1%	.317	<.001
BVH 2	56.4%	.417	<.001
OA 1	68.2%	.521	<.001
OA 2	63.9%	.428	<.001
OA 3	57.4%	.285	<.001
PN 1	63.3%	.512	<.001
PN 2	49.1%	.85	.091
PN 3	71.1%	.575	<.001
PN 4	47.3%	.310	<.001
Total (*n* = 16)	59.1%	.417	<.001

ID, subject identification; UVH, unilateral vestibular hypofunction; BVH, bilateral vestibular hypofunction; OA, older adult; PN, peripheral neuropathy.

### Individual-level agreement variability

Individual-level agreement ranged from poor to moderate across participants (Table 2). Eight participants demonstrated moderate agreement (*k* = 0.41–0.60), six demonstrated fair agreement (*k* = 0.21–0.40), and two demonstrated poor agreement (*k* ≤ 0.20). The highest individual agreement was observed for participant PN3 (*k* = 0.58, *P* < 0.001), while the lowest was observed for participant UVH4.

### Weighted kappa analysis

Weighted kappa analyses accounting for the magnitude of rating discrepancies yielded higher agreement estimates than unweighted kappa. Linear weighted kappa was *k* = 0.58 (*P* < 0.001), indicating moderate agreement. Quadratic weighted kappa was *k* = 0.74 (*P* < 0.001), indicating substantial agreement. Tables 3, 4 illustrate how the weighting differed for the linear weighting vs. the quadratic weighted kappa correlation by rating separation.

**Table 3:** Weighting assignments for each of the possible participant and physical therapist rating combinations for the linear weighted kappa correlation analysis.

Ratings		Participant rating				
		1	2	3	4	5
Physical therapist rating	1	1.00	0.75	0.50	0.25	0
	2	0.75	1.00	0.75	0.50	0.25
	3	0.50	0.75	1.00	0.75	0.50
	4	0.25	0.50	0.75	1.00	0.75
	5	0	0.25	0.50	0.75	1.00

**Table 4:** Weighting assignments for each of the possible participant and physical therapist rating combinations for the quadratic weighted kappa correlation analysis.

Ratings		Participant rating				
		1	2	3	4	5
Physical therapist rating	1	1.00	0.9375	0.75	0.4375	0
	2	0.9375	1.00	0.9375	0.75	0.4375
	3	0.75	0.9375	1.00	0.9375	0.75
	4	0.4375	0.75	0.9375	1.00	0.9375
	5	0	0.4375	0.75	0.9375	1.00

### Distribution of rating discrepancies

Of the 1,658 rating comparisons, 966 (58%) were exact matches between the participant numerical rating from the 5-point color-coded Likert-type balance scale and the clinician numerical ratings (Figure 1). Among the 692 comparisons with discrepancies, 587 (85%) differed by only one number separation (for example, PT rating = “1” and participant rating = “2”), 97 (14%) differed by two numbers separation (for example, PT rating = “5” and participant rating = “3”), and eight (1%) differed by three numbers separation. This pattern indicates that most disagreements were relatively minor, with substantial discrepancies (≥3 numbers separation) being rare.

At the individual level, nine participants tended to rate exercises as less difficult than clinicians rated the assistance provided (participant rating < clinician rating), while six participants tended to rate exercises as more difficult (participant rating > clinician rating). One participant (PN3) achieved exact agreement on 71% (64/90) of rating comparisons. Among the 26 discrepant ratings for PN3, all differed by only one number separation, with 12 instances where the clinician rated higher assistance and 14 instances where the participant rated higher difficulty.

## Discussion

This exploratory study provides preliminary evidence of moderate to substantial agreement between participant-perceived difficulty and clinician-assessed assistance levels during balance exercises. The Cohen's kappa coefficient and quadratic weighted kappa analysis suggest potential utility of verbal rating scales for assessing balance exercise difficulty. While these initial findings indicate participant feedback may inform individualized exercise progression, further validation in larger, more diverse samples is needed before widespread clinical implementation. Importantly, the current study did not directly assess functional outcomes, safety, or adherence; therefore, claims regarding improved efficacy in balance rehabilitation remain to be empirically tested.

This study is among the first to directly compare participant and clinician balance ratings in a structured rehabilitation program. The use of validated rating scales and a systematic progression framework enhanced the reliability and applicability of the findings. The inclusion of participants with diverse balance enhances the ecological validity of our findings, as clinicians typically treat heterogeneous patient populations. However, this heterogeneity also introduces variability in underlying mechanisms of balance control (sensory, motor, cognitive), which may differentially affect the relationship between perceived difficulty and required assistance. While our sample size did not afford the opportunity to conduct subgroup analyses, future studies should examine whether rating agreement differs systematically across diagnostic categories. Visual inspection of agreement patterns suggested no clear or consistent differences in agreement between participant and clinician ratings across balance exercise categories, which is theoretically grounded given that all exercises were performed in standing. A limitation of our study is the small sample size. Recruitment for the study was challenged by the commitment required by the participant to fulfill 23 sessions (18 training and 5 testing). This may have biased our sample into investigating people who may be more invested in improving their health and function. In addition to the time commitment, the other two factors that posed a problem to our recruitment was excluding people who had a history of a total joint replacement and excluding people whose MOCA score was <26 out of 30. Eight people interested in the study were excluded during the phone screen because of a history of a joint replacement and seven people were excluded because they had a MOCA <26 during the clinical screening.

Evidence suggests that many older adults and people with chronic neurological disorders may not receive adequately dosed physical therapy to achieve optimal outcomes (15–18). The American Board of Internal Medicine Foundation initiated a campaign called “Choosing Wisely” to put healthcare recommendation into practice (19) and in conjunction with this campaign, the American Physical Therapy Association released a list of five recommendations to provide evidence-based physical therapy (20). Physical therapists are urged not to prescribe under-dosed strength training programs for older adults and the recommendation to match the frequency, intensity, and duration of exercise to the individual's ability and goals was specifically stated.

Individualizing the difficulty of balance exercises may help address this gap. By tailoring difficulty levels to each participant's abilities, clinicians can provide exercises that are achievable yet sufficiently challenging, enhancing self-efficacy and supporting positive expectations. This approach aligns with challenge point theory, which posits that motor learning is maximized when tasks are neither too easy nor too difficult relative to the learner's skill level (21). In the context of balance training, adjusting difficulty individually may not only optimize motor learning but also promote engagement and confidence, potentially supporting greater adherence and functional gains over time.

Most of the research on exercise intensity has surrounded cardiovascular and resistance training exercise. While our study did not directly examine the relationship between exercise difficulty and functional outcomes, existing literature on exercise dosing (15–18) suggests that adequate intensity may be similarly important for balance training. Without standardized methods to quantify balance exercise difficulty, progression of exercises is difficult. Future research should investigate whether specific difficulty levels correspond with optimal functional outcomes. Additional efforts in establishing guidelines for balance programs that include dose recommendations using the FITT principle (frequency, intensity, time, type) is beneficial for healthcare providers and consumers (22). During the conception of this project, the intention was to create both an intense program that was individualized for each participant but that also followed a reproducible paradigm consistent with randomized control trial expectations. To do this, we followed progression rules designed by our study team which may have led to a prescription of balance exercises that were not difficult enough. While there is not a recommended difficulty level for balance programs, other investigators have speculated that balance training in their study lacked effectiveness due to insufficient intensity (23).

Discrepancies between participant and clinician ratings highlight psychological factors that may influence balance performance beyond physical capability alone. Participants who rated exercises as more difficult than observed by the physical therapist may have experienced reduced self-efficacy or heightened fear of falling, factors associated with activity avoidance and increased fall risk (24, 25). Fear of falling can also contribute to a self-reinforcing cycle of activity avoidance and functional decline (26). Conversely, participants who underestimated exercise difficulty may have limited awareness of their balance limitations, potentially increasing the risk of unsafe activity choices in daily life (27). These discrepancies underscore that perceived difficulty reflects both physical capability and psychological state, and that monitoring agreement between participant and clinician ratings may provide a clinically accessible indicator of these influences. Incorporating strategies to build confidence and align participants' perceptions with their actual abilities (e.g., such as performance accomplishments through mastery experiences, verbal encouragement, and reframing physiological responses to exercise) may increase engagement (28), enhance adherence to training (29), and optimize overall training outcomes (30). Future research should integrate validated psychological measures alongside participant and clinician ratings to better understand how discrepancies relate to self-efficacy, fear of falling, and balance training outcomes. Understanding these relationships could inform more comprehensive, person-centered approaches to balance exercise prescription that address both physical and psychological contributors to fall risk.

Several methodological features warrant consideration. First, while the physical therapist clinician ratings were finalized independently prior to being aware of participant ratings for each exercise, the clinician could have become aware of participant rating patterns across sessions through the progression planning process. Second, the ratings in our investigation served dual purposes, both to assess agreement between the participant and clinician, and to inform balance exercise progression. The dual role may have introduced circularity, as ratings influenced task selection in subsequent sessions and potentially constrained the range of future rating comparisons. Third, our conservative progression rules (requiring ratings <5 before advancing difficulty) may have limited exploration of higher difficulty levels. These features reflect the pragmatic nature of research embedded within clinical care; however, future studies should include independent rating sessions that do not affect treatment progression to eliminate potential circularity.

Additionally, while literature on task-specific training suggests its importance for neuroplasticity and motor learning (31–34), our study did not directly measure functional gains or assess whether task-specific exercises would have yielded different outcomes. The type of balance exercises included in our program may not have fully aligned with participants' individual functional goals. It is suspected that in the clinical setting, physical therapists and other rehabilitation clinicians will talk to a patient with a balance disorder about their goals and then incorporate those functional tasks into the training program. This is something that was not included in our training program and may have affected the clinical outcomes and quality of life measures. It has been recommended that rehabilitation incorporates task-specific training that includes activities that are typically performed in everyday life that are intrinsically and extrinsically meaningful (32). Our study participants may have been more engaged if the tasks were more relevant to their functional goals and perhaps further improvements in function and quality of life would have resulted.

The findings highlight the need for clear intensity guidelines in balance rehabilitation, similar to those for aerobic and resistance exercises. Incorporating participant feedback into exercise planning may enhance individualized care, although its impact on rehabilitation outcomes was not directly assessed. An important next step is to determine whether specific difficulty levels during balance training predict improved functional outcomes, which would enable clinicians to target optimal challenge zones for individual patients. Such investigations also should investigate optimal balance training intensity and explore task-specific exercise designs to establish evidence-based progression guidelines. Additionally, future studies with larger samples should examine whether rating agreement differs systematically across diagnostic categories, as distinct mechanisms of balance impairment may influence the relationship between perceived difficulty and required assistance. Understanding condition-specific patterns could inform tailored application of rating systems in clinical practice.

## Conclusion

This exploratory study examined agreement between participant-perceived balance difficulty and clinician-rated assistance during progressive balance training in individuals with neurological and balance disorders. The findings demonstrated moderate to substantial agreement between participant and clinician ratings, suggesting that participant feedback may provide a clinically accessible indicator of exercise difficulty in balance rehabilitation.

However, several limitations temper confidence in clinical application. The study's small, single-site sample and single physical therapist limit generalizability and preclude assessment of inter-rater reliability across clinicians. Methodologically, ratings served dual purposes—both assessing agreement and informing progression—which may have introduced circularity. Additionally, the study did not directly measure functional outcomes, safety, or adherence; therefore, whether incorporating participant ratings into exercise prescription improves rehabilitation outcomes remains to be empirically demonstrated.

Future research should validate these findings in larger, multi-site samples with multiple clinicians to establish broader applicability and inter-rater reliability. Critically, studies examining whether specific difficulty levels predict improved functional outcomes would enable evidence-based targeting of optimal challenge zones. Additionally, investigating the integration of task-specific exercises and validated psychological measures (e.g., self-efficacy, fear of falling) alongside difficulty ratings would support more comprehensive, person-centered approaches to balance rehabilitation. Establishing standardized methods for quantifying and progressing balance exercise difficulty remains a critical need in neurological rehabilitation.

## Data Availability

The raw data supporting the conclusions of this article will be made available by the authors, without undue reservation.
